# Long-term monitoring of fatty acid oxidation defects: results from a MetabERN survey

**DOI:** 10.1186/s13023-024-03024-0

**Published:** 2024-01-20

**Authors:** Marit Schwantje, Sarah C. Grünert, Sabine A. Fuchs

**Affiliations:** 1 Department of Metabolic Diseases, Wilhelmina Children’s Hospital, University Medical Centre Utrecht, Lundlaan 6, 3584 EA Utrecht, The Netherlands; 2 Department of General Paediatrics, Adolescent Medicine and Neonatology, Faculty of Medicine, University Medical Centre, Mathildenstraße 1, 79106 Freiburg, Germany

**Keywords:** Long-chain fatty acid oxidation defects (LCFAOD), Long-term monitoring, Clinical follow-up, Europe-wide, Newborn screening

## Abstract

**Background:**

Implementation of long-chain fatty acid oxidation defects (LCFAOD) in newborn screening (NBS) programs allows for pre-symptomatic diagnosis and treatment. The long-term natural history of NBS LCFAOD patients is largely unknown and may differ from clinically diagnosed pre-NBS patients. This complicates long-term monitoring of LCFAOD and may cause high monitoring variability. To gain insight in current clinical practice, we performed a web-based questionnaire among all metabolic members of the European Reference Network for Hereditary Metabolic Disorders (MetabERN).

**Results:**

Thirty-seven colleagues representing at least 35 European metabolic centres shared their experience and results were discussed at the European Metabolic Group (EMG) meeting 2022. The centres concurred in many aspects of long-term monitoring of LCFAOD including the frequency of clinical visits, determination of laboratory parameters, cardiac monitoring and retinopathy screening. Main discrepancies comprised hepatic imaging, glucose monitoring and electrophysiological investigations.

**Conclusions:**

Discrepancies may reflect differences in local availability of monitoring tools, the inclusion of LCFAOD in NBS programs as well as differences in local genotypes and phenotypes. Because monitoring strategies are largely based on the natural disease course of clinically identified patients, there might be over-monitoring of some NBS patients. Nevertheless, we advocate long-term monitoring because resulting information is essential to further characterize the natural disease course, develop evidence-based guidelines and provide a basis for evaluation of future therapies.

**Supplementary Information:**

The online version contains supplementary material available at 10.1186/s13023-024-03024-0.

## Background

Long-chain fatty acid oxidation defects (LCFAOD) represent a group of inborn errors of metabolism characterized by hepatic, muscular and cardiac symptoms as these organs strongly depend on fatty acid oxidation for energy generation. In some defects also neuropathy and/or retinopathy occur as long-term complications [1, 2]. Patients are at risk of metabolic decompensation especially during situations with high energy expenditure such as febrile illnesses and exercise, or during fasting. LCFAOD present with a wide clinical heterogeneity, ranging from a severe phenotype with neonatal cardiomyopathy to milder phenotypes with only muscle symptoms developing during adolescence [1, 2]. Some patients may even remain asymptomatic [3].

Newborn screening (NBS) programs have been implemented in many countries worldwide, allowing for pre-symptomatic identification of patients and early initiation of dietary treatment [4, 5]. However, at the time of the diagnosis, it is unknown whether and to what extent these patients will develop symptomatic disease. In addition to the wide disease spectrum, the disease course of pre-symptomatically diagnosed NBS patients with early treatment initiation is unknown and may differ from patients diagnosed based on clinical symptoms, the pre-NBS LCFAOD population [3]. Uncertainty regarding the disease course of this new patient cohort makes it difficult to define an appropriate long-term monitoring regimen. Consequently, long-term monitoring may vary highly between centres, and there are still many questions regarding the necessity, frequency and clinical consequences of different follow-up investigations.

To gain insight into the current clinical practice, we performed a questionnaire study on long-term monitoring approaches for LCFAOD in different European metabolic centres with the ultimate aim to improve follow-up and care for LCFAOD patients.

## Methods

A web-based questionnaire on long-term monitoring of LCFAOD was designed and shared using Google Forms. The questionnaire was distributed to members of the European Reference Network for Rare Hereditary Metabolic Disorders (MetabERN) and participants of a workshop at the European Metabolic Group (EMG) meeting in Innsbruck in May 2022, where preliminary results were discussed among the 25 attending experts. One response per centre was requested. The questionnaire mostly consisted of multiple choice questions and addressed several aspects of long-term monitoring of LCFAOD, including frequency and methods used to follow up on nutritional history, risk of hypoglycaemia, development, cardiomyopathy and rhythm disorders, myopathy, hepatic manifestations, and peripheral neuropathy and pigmentary retinopathy in case of long-chain 3-hydroxyacyl-CoA dehydrogenase deficiency (LCHADD) and mitochondrial trifunctional protein deficiency (MTPD) (see Additional file 1 for an overview of included questions). We excluded questionnaires with over 50% missing answers (n = 3) and duplicate cases (n = 1). Any remaining missing data were handled by pairwise exclusion.

## Results

### Questionnaire respondents

**Fig. 1:**
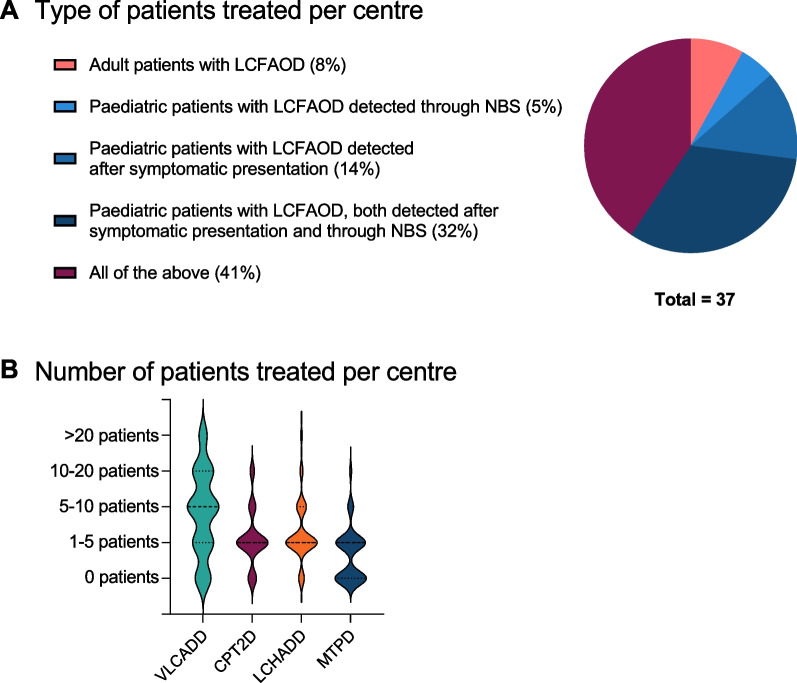
**A** Overview of the qualification of the respondents showing the type of patients treated per centre. **B** Overview of the number of patients with each LCFAOD treated in the different centres. *LCFAOD* Long chain fatty acid oxidation defects, *NBS* Newborn screening, *VLCADD* Very long-chain acyl-CoA dehydrogenase deficiency, *CPT2D* Carnitine palmitoyltransferase 2 deficiency, *LCHADD* Long-chain 3-hydroxyacyl-CoA dehydrogenase deficiency, *MTPD* Mitochondrial trifunctional protein deficiency

Thirty-seven metabolic specialists (97% physicians, 3% metabolic dietician) representing at least 35 European metabolic centres from 16 different countries shared their experience (Additional file 2). For one respondent, information on nationality and metabolic centre was missing. Most respondents (51%, 19/37) were involved only in paediatric care: 32% (12/37) for paediatric patients diagnosed by NBS and after symptomatic presentation, 5% (2/37) only for paediatric patients diagnosed by NBS and 14% (5/37) only for paediatric patients diagnosed after symptomatic presentation. 41% (15/37) was involved in both paediatric and adult care, whereas 8% (3/37) only in adult care (Fig. 1A). Figure 1B shows the number of LCFAOD patients treated in the different metabolic centres. Very long-chain acyl-CoA dehydrogenase deficiency (VLCADD) patients were most commonly seen. Most centres only treated 1 to 5 patients with carnitine palmitoyltransferase 2 deficiency (CPT2D) and LCHADD, and no patients with MTPD.

### Frequency of follow-up

Frequency of outpatient follow-up decreased with age from every 1 to 3 months during infancy to every 3 to 6 months in toddlers, every 3 to 12 months (mostly every 6 months) in older children aged 4–12 years and every 6 to 12 months from 12 years onwards (Additional file 3). The frequency of follow-up in different age groups was homogeneous among the different metabolic centres.

### Nutritional follow-up

Nutritional follow-up was performed during every follow-up appointment at the outpatient clinics according to 76% of respondents (28/37), every 6 months according to 8% (3/37) and yearly according to 14% (5/37). One respondent (3%, 1/37) did not perform nutritional follow-up according to a standard schedule. Essential fatty acids were routinely checked in all patients by 47% (7/15) of the respondents and only in case of an MCT enriched, LCT restricted diet by 20% (3/15).

### Acylcarnitine profiles

86% (32/37) of respondents reported to perform acylcarnitine profiling as part of the routine follow-up. The remaining respondents only measured acylcarnitine profiles during the diagnostic process. 81% (25/31) reported to adjust the patient’s dietary regimen based on the acylcarnitine levels.

The question whether carnitine supplementation was recommended emphasized the existing controversy regarding carnitine supplementation in LCFAOD. Of 36 respondents, 64% (23/36) answered that they did not recommend carnitine supplementation, whereas 36% (13/36) did, mostly in case of a proven carnitine deficiency.

### Glucose monitoring

68% of respondents (25/37) reported to routinely measure glucose levels at the outpatient clinics. The question regarding glucose monitoring at home was answered by 25 respondents, with half (52%, 13/25) advising glucose monitoring at home, generally for specific cases. For example, one respondent advised glucose monitoring at home only for certain LCFAOD (LCHADD, VLCADD), and ten respondents advised glucose monitoring at home only if a patient had experienced a hypoglycaemic event.

### Muscle symptoms

All but one centre reported to measure creatine kinase (CK) levels as part of the routine follow-up (97%, 36/37). Clearly less respondents measured myoglobin levels in serum (routinely: 19%, 7/37; in case of suspected illness/rhabdomyolysis: 46%, 17/37). 19% (7/36) reported to advise urinary dipsticks to measure myoglobin levels to monitor rhabdomyolysis at home. 17% (6/35) reported to perform imaging of the muscles, either by muscle ultrasound or magnetic resonance imaging (MRI) (Additional file 4A).

### Exercise testing

Five respondents (14%, 5/37) had experience with exercise testing in LCFAOD patients. One of these five had only performed exercise testing once in an LCHADD patient [6].

Two respondents performed both a maximal and an endurance exercise test, two only a maximal test and one only an endurance test. Four indicated that the results could have clinical consequences (e.g. changes in diet, exercise recommendations).

### Cardiac follow-up

39% of the respondents (14/36) reported to measure cardiac markers as part of the routine follow-up, while half (53%, 19/36) only measured cardiac markers in case of cardiac signs or symptoms. Among those, three also determined these markers in case of severe rhabdomyolysis. Two respondents (6%, 2/36) reported to never measure cardiac markers, and one only measured cardiac markers after consultation with a cardiologist. In routine follow-up, the most frequently measured cardiac markers were (N-terminal pro) B-type natriuretic peptide ((NT pro)BNP, 79%, 11/14) and troponins (57%, 8/14) (Additional file 4B).

84% of the respondents (31/37) reported to perform electrocardiograms (ECG) as part of the routine follow-up. 8% (3/37) additionally performed 24-h holter monitoring. 14% (5/37) performed ECG or 24-h-holter follow-up only in case of cardiac symptoms and 3% (1/37) only in case of a severe LCFAOD phenotype. 92% (34/37) reported to perform an echocardiogram as part of the routine follow-up, while the remainder only performed echocardiograms in case of cardiac symptoms.

Most paediatricians started cardiac monitoring (ECG and echocardiograms) directly after diagnosis or in the first year of life (76% (16/21) for ECG and 88% (23/26) for echocardiograms). The remaining respondents reported to start cardiac follow-up later in life (3–10 years of age). Half of the respondents monitored cardiac function once a year (53% (17/32) for ECG, 52% (17/33) for echocardiogram).

In most of the remaining respondents, cardiac follow-up was performed less frequently (once every 2 or 3 years, especially in patients with milder phenotypes), depending on disease severity or patient’s age, or not according to a standard schedule. A minority monitored cardiac function more frequently (3% (1/32) for ECG, 8% (3/33) for echocardiogram).

### Hepatic follow-up

Almost all respondents (97%, 36/37) reported to measure liver enzymes and/or liver function during routine follow-up. All respondents measured aspartate aminotransferase (ASAT) and alanine aminotransferase (ALAT), 78% (28/36) gamma-glutamyltransferase (gGT), 75% (27/36) albumin levels and 61% (22/36) alkaline phosphatase. Coagulation parameters were measured less frequently (44%, 16/36) (Additional file 4C).

Hepatic ultrasounds were performed routinely by the majority of the respondents (54%, 20/37), most of them (84%, 16/19) starting after confirmation of the diagnosis or in the first year of life. 40% of the respondents (8/20) reported to perform a hepatic ultrasound every year, and 25% (5/20) every other year. For others, the frequency depended on the patient’s age (10%, 2/20), previous findings (5%, 1/20), or frequency was not determined by a standard time schedule (20%, 4/20).

### General clinical follow-up

Standardized developmental follow-up was reported by 51% of centres (18/35). Standardized assessment of quality of life was performed routinely by 30% (11/37).

### Long-term complications of LCHADD and MTPD

#### Retinopathy

Thirty-one respondents treated one or more LCHADD or MTPD patients. Almost all of them (94%, 29/31) reported to perform fundoscopy as part of the routine follow-up of LCHADD and MTPD patients, with a frequency of once a year in 55% (16/29), once every 2 years in 10% (3/29), once every 3 years in 7% (2/29). In the remaining, the frequency was not according to a standard schedule, depended on the patient’s age or on eye involvement/disease severity. Respondents started fundoscopy screening in the first or second year of life (65%, 17/26), or between the age of 2 to 10 years (35%; 9/26).

Two respondents only performed fundoscopy in case of signs or symptoms of retinopathy. Upon signs/symptoms of retinopathy, most respondents performed a fundoscopy yearly (65%, 20/31) or once every 2 years (6%, 2/31). In the remaining, the frequency of follow-up was not according to a standard schedule, depended on eye involvement/disease severity, or followed the advice of an ophthalmologist.

Electroretinography (ERG) was usually not performed as part of the routine follow-up, but when performed (30%, 9/30), screening started between 1 to 10 years of age, with a frequency of once a year (22%, 2/9), once every 2 years (11%, 1/9), 3 years (22%, 2/9), or not according to a standard time schedule (44%, 4/9). In case of signs or symptoms or retinopathy, most respondents (42%, 11/26) did not have a standard schedule, performed an ERG every year (31%, 8/26) every other year (4%, 1/26) or followed the advice of an ophthalmologist (23%, 6/26) (Additional file 4D).

### Neuropathy

Most respondents (65%, 20/31) performed electrophysiological examinations (nerve conduction studies (ENG) and/or electromyography (EMG)) only in patients with clinical signs of neuropathy. Electrophysiological examinations were part of the routine follow-up in 23% of respondents (7/31). These respondents mostly performed only ENG (86%, 6/7) from an age between 1 and 6 years onwards, once a year in 14% (1/7), once every 2 years in 43% (3/7) or not according to a standard time schedule 43% (3/7).

## Discussion

This questionnaire study provides an overview of the current approach to long-term monitoring of LCFAOD in Europe. Although the questionnaire revealed some differences between metabolic centres, general aspects of follow-up such as cardiac monitoring and frequency of follow-up were relatively comparable, despite potential differences between NBS and pre-NBS populations and the absence of evidence-based guidelines and monitoring recommendations.

The questionnaire focused on multiple aspects of long-term monitoring. The additional value of and the reasoning behind several routine follow-up investigations was critically discussed among metabolic experts during the EMG workshop.

Follow-up frequency at the outpatient clinics was relatively homogeneous among the European metabolic centres. Follow-up frequency generally decreased with age, from every 1 to 3 months during infancy to every 6 to 12 months from 12 years onwards.

Acylcarnitine profiling was part of the follow-up in 86% of the respondents, and most of them (81%) adjusted the dietary regimen accordingly. Details on how the dietary regimen was adjusted, which criteria were used and how this influenced the acylcarnitine profile and clinical condition were not available. Analysis of these data would help to determine the role of acylcarnitine profiling in disease management.

All but one European metabolic centre measured CK levels as part of the routine follow-up, while plasma and urinary myoglobin concentrations and muscle MRIs were part of routine follow-up in only a few centres. EMG workshop participants indicated that CK concentrations were considered the most important biomarker to complement the history of muscle symptoms and to assess muscle involvement. In case of severe muscle symptoms or coloured urine suspicious for rhabdomyolysis, they would advise to see the patient in the hospital, regardless of the results of the home measurement of urinary myoglobin levels.

Cardiac monitoring was generally part of the routine follow-up from diagnosis or the first year of life onwards. The majority of respondents monitored cardiac function with ECG and echocardiogram yearly in asymptomatic patients, and intensified cardiac monitoring based on disease severity and symptoms. Screening of asymptomatic individuals was deemed important as cardiomyopathy can occur at any age [7, 8]. Severe weight loss may both cause and reflect cardiac deterioration and therefore warrants cardiac evaluation. For carnitine-acylcarnitine translocase (CACT) deficiency and MTPD patients with fever, cardiac monitoring was specifically recommended based on the clinical experience of the workshop participants with increased risk of cardiomyopathy in these patients during acute illness.

Glucose concentrations were routinely analysed during outpatient clinic visits, but none of the workshop participants had identified asymptomatic hypoglycaemia under healthy conditions. This renders random glucose analysis questionable. Glucose monitoring at home was recommended by half of the respondents under certain conditions. Although glucose monitoring at home may provide reassurance, it may also cause a ‘false sense of security’. If a patient is at risk of developing hypoglycaemia, (s)he should present in the hospital rather than await hypoglycaemia at home. Moreover, home monitoring may lead to over-monitoring and may be complicated by technical issues. The workshop discussion led to the conclusion that glucose monitoring at home should not be part of the routine monitoring of LCFAOD patients and only be advised in individual cases when deemed truly beneficial.

Hepatic involvement was monitored by measuring liver enzymes and/or liver function in most centres. Half of the respondents additionally performed hepatic ultrasound, mainly to screen for possible liver abnormalities such as steatosis. However, abnormalities were seldom found in asymptomatic patients and when steatosis was observed, it usually had no implication for clinical management. Therefore, we concluded that hepatic ultrasound during the routine follow-up mainly serves to gain insight in the hepatic phenotype of LCFAOD.

Although currently only performed in the routine follow-up in a few centres in Europe, exercise testing can be used to empower patients by documenting safety of exercise. Regular physical activity, within safe boundaries for individual patients, should be encouraged to improve both social and physical health. Moreover, exercise testing can be used to monitor exercise (in)tolerance as a characteristic of the natural disease course and to evaluate treatment response. In the future, exercise testing may become more common in the follow-up of LCFAOD.

In LCHADD and MTPD patients, retinopathy was typically monitored by using fundoscopy as part of the routine follow-up of asymptomatic patients, whereas neuropathy was mainly monitored by neurophysiologic investigations (ENG/EMG) in patients with neuropathic symptoms. Besides fundoscopy and ERG, optical coherence tomography (OCT) was mentioned as a possible screening method for retinopathy. It is debatable whether retinopathy and neuropathy screening should be performed on a routine basis because of the absence of adequate treatment. Some patients are reluctant towards the regular confirmation of disease progression. Nevertheless, recent evidence [9] suggests that LCHADD- and MTPD-related neuropathy may be (partly) reversible, putatively in response to dietary interventions at a very early stage of the disease, emphasizing the need for early diagnosis. Additionally, information on the severity of these complications is needed for optimal supportive patient care. Looking from a research perspective, collected data helps to gain insight into the natural course, serve as a basis for the evaluation of effects of possible treatments and may help to clarify the underlying pathophysiology of LCHADD- and MTPD-related retinopathy and neuropathy.

Standardized assessment of quality of life was not part of the routine follow-up in most centres*.* So far, there are no standardized tools to assess patient-reported outcomes in LCFAOD patients. The development of instruments that measure what matters most to patients with LCFAOD will not only be important for clinical care but also as outcome measures for future clinical trials [10].

**Fig. 2:**
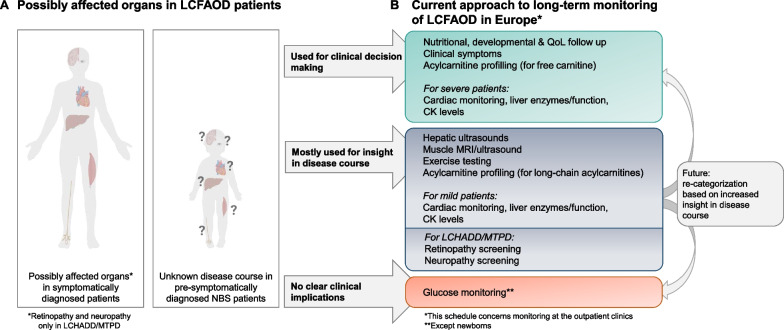
Overview of the possibly affected organs in LCFAOD (**A**) and the current approach to long-term monitoring of LCFAOD in Europe (**B**). Follow-up procedures are categorized in three groups: 1 used for clinical decision making (upper box), 2 mostly used to gain insight in disease course (middle box) and 3 without clear clinical implications (lower box). Future insight in disease course of the new NBS-population may allow re-categorization of follow-up procedures into relevant for clinical decision making or without clear clinical implications. *LCFAOD* Long chain fatty acid oxidation defects, *LCHADD* Long-chain 3-hydroxyacyl-CoA dehydrogenase deficiency, *MTPD* Mitochondrial trifunctional protein deficiency, *NBS* Newborn screening, *QoL* Quality of life, *CK* Creatine kinase

The questionnaire study included European metabolic centres treating different groups of LCFAOD patients, varying from only adult patients to symptomatically and/or NBS-diagnosed paediatric patients. The natural history of the NBS LCFAOD cohort may well differ from the pre-NBS population, diagnosed based on clinical symptoms [3]. Nevertheless, follow-up procedures are based on the putatively more severe pre-NBS population characteristics. This may lead to extensive and invasive long-term monitoring in pre- or asymptomatic patients without supporting evidence. Precise long-term monitoring is however required to gain insight into the clinical disease course of this new patient cohort. Based on the findings of this study and current knowledge regarding NBS patients’ disease course [3], we categorized currently performed follow-up procedures into three groups: (1) used for clinical decision making, (2) mostly used to gain insight into disease course and (3) without clear clinical implications (Fig. 2). Procedures were categorized as ‘mostly used to gain insight into disease course’ (Fig. 2, middle box), when abnormalities were infrequent (e.g. cardiac abnormalities in mild NBS patients), without treatment implications (e.g. retinopathy screening, muscle MRI), or not (yet) broadly used (e.g. exercise testing). Further disease monitoring will re-categorize procedures into relevant for clinical decision making (upper box), or without clear clinical implications (lower box). Data from the Unified European Registry for Inherited Metabolic Disorders (U-IMD) [11] will further add to evidence-based guideline development.

In conclusion, metabolic centres in Europe concur in their long-term follow-up of LCFAOD for general aspects, including follow-up frequency, CK and liver enzyme analysis, cardiac monitoring, and retinopathy screening. However, there are discrepancies in other aspects such as hepatic ultrasound, glucose monitoring, exercise testing, and electrophysiological investigations. These differences may reflect differences in local availability of monitoring tools, differences in local genotypes and clinical phenotypes, but also differences in the expected (milder) clinical phenotype of the NBS-population. Although there might be over-monitoring in NBS patients, precise and standardized long-term monitoring is necessary to gain insight in the natural disease course of this new population and to create a basis for systematic evaluation of follow-up procedures, develop evidence-based guidelines and provide a framework for future therapies.

### Supplementary Information

**Additional file 1:** An overview of the questions and answer options included in the questionnaire on long-term monitoring of LCFAOD.

**Additional file 2:** An overview of the included countries and the number of included metabolic centres per country.

**Additional file 3:** Overview of the reported follow-up frequencies in infants (**A**), toddlers (**B**), children (**C**), adolescents and adults (**D**).

**Additional file 4:** Reported muscle, cardiac, hepatic, retinopathy and neuropathy monitoring in the different centres.

## Data Availability

The raw data, not described in detail in this article, will be accessible through DataverseNL (10.34894/LEWTBK).

## Abbreviations

LCFAOD: Long-chain fatty acid oxidation defects
NBS: Newborn screening
MetabERN: European Reference Network for Rare Hereditary Metabolic Disorders
EMG: European Metabolic Group
LCHADD: Long-chain 3-hydroxyacyl-CoA dehydrogenase deficiency
MTPD: Mitochondrial trifunctional protein deficiency
VLCADD: Very long-chain acyl-CoA dehydrogenase deficiency
CPT2D: Carnitine palmitoyltransferase 2 deficiency
ENG: Electroneurography
EMG: Electromyography
ERG: Electroretinography
