# Supplementary material for: Long-term monitoring of fatty acid oxidation defects: results from a MetabERN survey
Source: Orphanet J Rare Dis. 2024 Jan 20;19:21. doi: 10.1186/s13023-024-03024-0 (PMC10800038; doi:10.1186/s13023-024-03024-0)
Supplement: Supplementary file 1 — Additional file 1: An overview of the questions and answer options included in the questionnaire on long-term monitoring of LCFAOD. [file 13023_2024_3024_MOESM1_ESM.pdf]

Additional File 1 – An overview of the questions and answer options included in the questionnaire on long-term monitoring of LCFAOD

	Question	Answer format	Multiple choice options*
**1**	Are you a ...?	Multiple choice	Medical specialist; Laboratory specialist; Nutrition specialist; Researcher; Exercise physiologist; Other
**2**	What is the metabolic centre you are working at?	Open	
**3**	Can we have your email address to contact you for further questions? If yes, what is your email address?	Open	
**4**	Which patient group(s) do your answers in this questionnaire concern?	Multiple choice	Adult patients with LCFAOD; Paediatric patients with LCFAOD detected through newborn screening; Paediatric patients with LCFAOD detected after symptomatic presentation; Paediatric patients with LCFAOD, both detected after symptomatic presentation and through newborn screening; All of the above; Other
**5**	How many patients with VLCAD deficiency are treated in your medical center?	Multiple choice	0 patients; 1-5 patients; 5-10 patients; 10-20 patients; >20 patients
**6**	How many patients with CTP2 deficiency are treated in your medical center?	Multiple choice	0 patients; 1-5 patients; 5-10 patients; 10-20 patients; >20 patients
**7**	How many patients with LCHAD deficiency are treated in your medical center?	Multiple choice	0 patients; 1-5 patients; 5-10 patients; 10-20 patients; >20 patients
**8**	How many patients with MTP deficiency are treated in your medical center?	Multiple choice	0 patients; 1-5 patients; 5-10 patients; 10-20 patients; >20 patients
**9**	What is the frequency of your routine follow-up of patients with VLCAD deficiency in the outpatient clinic in INFANCY (0-1 years)? Approximately...	Multiple choice	Every month; Every three months; Every six months; Other
**10**	What is the frequency of your routine follow-up of patients with CPT2 deficiency in the outpatient clinic in INFANCY (0-1 years)? Approximately...	Multiple choice	Every month; Every three months; Every six months; Other
**11**	What is the frequency of your routine follow-up of patients with LCHAD deficiency in the outpatient clinic in INFANCY (0-1 years)? Approximately...	Multiple choice	Every month; Every three months; Every six months; Other
**12**	What is the frequency of your routine follow-up of patients with MTP deficiency in the outpatient clinic in INFANCY (0-1 years)? Approximately...	Multiple choice	Every month; Every three months; Every six months; Other
**13**	What is the frequency of your routine follow-up of patients with VLCAD deficiency in the outpatient clinic for TODDLERS (1-3 years)? Approximately...	Multiple choice	Every month; Every three months; Every six months; Every year; Other
**14**	What is the frequency of your routine follow-up of patients with CPT2 deficiency in the outpatient clinic for TODDLERS (1-3 years)? Approximately...	Multiple choice	Every month; Every three months; Every six months; Every year; Other
**15**	What is the frequency of your routine follow-up of patients with LCHAD deficiency in the outpatient clinic for TODDLERS (1-3 years)? Approximately...	Multiple choice	Every month; Every three months; Every six months; Every year; Other
**16**	What is the frequency of your routine follow-up of patients with MTP deficiency in the outpatient clinic for TODDLERS (1-3 years)? Approximately...	Multiple choice	Every month; Every three months; Every six months; Every year; Other
**17**	What is the frequency of your routine follow-up of patients with VLCAD deficiency in the outpatient clinic for CHILDREN (4-12 years)? Approximately...	Multiple choice	Every month; Every three months; Every six months; Every year; Other
**18**	What is the frequency of your routine follow-up of patients with CPT2 deficiency in the outpatient clinic for CHILDREN (4-12 years)? Approximately...	Multiple choice	Every month; Every three months; Every six months; Every year; Other
**19**	What is the frequency of your routine follow-up of patients with LCHAD deficiency in the outpatient clinic for CHILDREN (4-12 years)? Approximately...	Multiple choice	Every month; Every three months; Every six months; Every year; Other
**20**	What is the frequency of your routine follow-up of patients with MTP deficiency in the outpatient clinic for CHILDREN (4-12 years)? Approximately...	Multiple choice	Every month; Every three months; Every six months; Every year; Other
**21**	What is the frequency of your routine follow-up of patients with LCFAOD in the outpatient clinic for ADOLESCENTS (12-18 years)? Approximately...	Multiple choice	Every month; Every three months; Every six months; Every year; Other
**22**	What is the frequency of your routine follow-up of patients with LCFAOD in the outpatient clinic for ADULTS (>18 years)? Approximately...	Multiple choice	Every month; Every three months; Every six months; Every year; Other
**23**	Nutritional follow-up: how often is diet evaluated and are nutrients and dietary calories calculated?	Multiple choice	During every routine follow-up appointment at the outpatient clinics; Every six months; Every year; Other
**24**	Is standardized developmental follow-up (IQ testing) part of your routine follow-up?	Multiple choice	Yes; No
**25**	Is standardized assessment of quality of life part of your routine follow-up?	Multiple choice	Yes; No
**26**	Is the measurement of the acylcarnitine profile part of routine follow-up?	Multiple choice	Yes; No, the acylcarnitine profile is only measured to diagnose the disease

**27**	If you see a rise in metabolic disease markers, do you adapt the patient's dietary regimen?	Multiple choice	Yes, in all patients; Yes, but only in patients with an LCT restricted, MCT supplemented diet; No
**28**	Do you recommend carnitine supplementation to patients with LCFAOD?	Multiple choice	Yes; No
**29**	When do you recommend carnitine supplementation? When free carnitine levels are below …	Open	
**30**	Is the measurement of creatinine kinase (CK) part of routine follow-up?	Multiple choice	Yes, CK levels are measured as part of follow-up; No, CK levels are only measured in case of illness or suspected rhabdomyolysis; Other
**31**	Is the measurement of serum myoglobin part of your follow-up?	Multiple choice	Yes, serum myoglobin is measured as part of routine follow-up; No, serum myoglobin is only measured in case of illness or suspected rhabdomyolysis; No, serum myoglobin is never measured during follow-up; Other
**32**	Do you recommend your patients to use urine dipsticks for myoglobin to monitor rhabdomyolysis at home?	Multiple choice	Yes; No
**33**	Do you perform muscle imaging by performing an muscle ultrasound of MRI?	Multiple choice	Yes; No
**34**	Are there any other tests you perform in the routine follow-up of myopathic symptoms? If yes, what other tests do you perform?	Open	
**35**	Do you perform exercise tests in patients with LCFAOD?	Multiple choice	Yes; No
**36**	Would you be willing to share your exercise test protocol in order to evaluate different protocols?	Multiple choice	Yes; No
**37**	What type of patients with LCFAOD are tested?	Multiple choice	Patients with VLCAD deficiency; Patients with CPT2 deficiency; Patients with MTP deficiency; patients with LCHAD deficiency
**38**	Do you perform a maximal exercise test?	Multiple choice	Yes; No
**39**	Do you perform an endurance exercise test?	Multiple choice	Yes; No
**40**	If an endurance exercise test is performed, how long does the endurance exercise test take and at what intensitiy is the exercise performed?	Open	
**41**	The frequency of exercise testing during follow-up is...	Multiple choice	Once a year; Once every two years; Not according to a standard schedule; Depending on patient's age; Other
**42**	What are the clinical consequences of the results of the exercise test? For example, do the results influence individual dietary or exercise recommendations?	Open	
**43**	Is the measurement of cardiac markers (e.g. troponins) part of routine follow-up?	Multiple choice	Yes, cardiac markers are measured as part of routine follow-up; Yes, cardiac markers are measured as part of routine follow-up; No, cardiac markers are never measured; Other
**44**	Which cardiac markers are measured during routine follow-up?	Multiple choice	Troponins; NT-proBNP; proBNP; CK-MB; Other
**45**	Is the performance of a standard elektrocardiogram (ECG) and/or 24-hour holter monitoring part of routine follow-up?	Multiple choice	Yes, we perform a standard electrocardiogram; Yes, we perform 24-hour holter monitoring; Yes, we perform both a standard ECG and 24-hour holter monitoring; No, these investigations are only performed in case of cardiac symptoms; Other
**46**	At what age (in years) does the performance of ECG and/or 24-hour holter monitoring as part of routine follow-up start?	Open	
**47**	The frequency an ECG and/or 24 hour holter monitoring is performed during routine follow-up of asymptomatic patients is...	Multiple choice	Once every six months; Once a year; Once every two years; Not according to a standard schedule; Depending on patient's age; Other
**48**	Is the performance of an echocardiogram part of routine follow-up?	Multiple choice	Yes; No, an echocardiogram is only performed in case of cardiac symptoms
**49**	At what age does the performance of echocardiograms during routine follow-up start?	Open	
**50**	The frequency an echocardiogram is performed during routine follow-up is…	Multiple choice	Once every six months; Once a year; Once every two years; Once every three years; Not according to a standard schedule; Depending on patient's age; Other
**51**	Are there any other tests you perform in the follow-up of cardiac symptoms? If yes, what other tests do you perform?	Open	
**52**	Is the measurement of glucose levels part of routine follow-up?	Multiple choice	Yes, glucose levels are measured as part of routine follow-up; No, glucose levels are only measured in case of illness or symptoms of hypoglycaemia
**53**	Do you recommend blood glucose monitoring at home to your patients?	Multiple choice	Yes, in case a patient has presented with hypoglycemia; Yes, in case a patient has presented with hypoglycemia; Yes, but only in certain long-chain fatty acid oxidation disorders (for example only in patients with MTP deficiency); No, never; Other

**54**	I recommend blood glucose monitoring at home for patients with …	Multiple choice	VLCAD deficiency; MTP deficiency; LCHAD deficiency; CPT2D deficiency
**55**	Is the measurement of liver enzymes (e.g. ASAT, ALAT) or liver function (e.g. aPTT) part of routine follow-up?	Multiple choice	Yes, the measurement of liver enzymes is part of routine follow-up; No, liver enzymes and function are only measured in case of illness or symptoms of hepatic manifestation
**56**	Which hepatic markers are measured during routine follow-up?	Multiple choice	Aspartate aminotransferase (ASAT); Alanine-aminotransferase (ALAT); Gamma glutamyl transferase (gamma GT); Alkaline phosphatase; Activated Partial Thromboplastin Time (aPTT) and prothrombin time (PT); Albumin level; Other
**57**	Which hepatic markers are measured during illness or signs of hepatic manifestation?	Multiple choice	Aspartate aminotransferase (ASAT); Alanine-aminotransferase (ALAT); Gamma glutamyl transferase (gamma GT); Alkaline phosphatase; Activated Partial Thromboplastin Time (aPTT) and prothrombin time (PT); Albumin level; Other
**58**	Is the performance of a hepatic ultrasound part of routine follow-up?	Multiple choice	Yes; No, a hepatic ultrasound is only performed in case of hepatic manifestation
**59**	At what age does the performance of hepatic ultrasound during routine follow-up start?	Open	
**60**	The frequency of performing a hepatic ultrasound during routine follow-up is …	Multiple choice	Once a year; Once every two years; Not according to a standard schedule; Depending on patient's age; Other
**61**	Are there any other tests you perform in the follow up of hepatic disease manifestation in patients? If yes, what other tests do you perform?	Open	
**62**	The patient group I am involved with includes patients with MTP and/or LCHAD deficiency	Multiple choice	Yes; No
**63**	Is the performance of a fundoscopy part of routine follow-up?	Multiple choice	Yes; No, a fundoscopy is only performed in case a patient develops signs of retinopathy; No, a fundoscopy is never performed.
**64**	The frequency a fundoscopy is performed in case a patient has developed signs of retinopathy, is …	Multiple choice	Once a year; Once every two years; Once every three years; Not according to a standard schedule; Depending on patient's age; Other
**65**	At what age does the performance of fundoscopies during routine follow-up start?	Open	
**66**	The frequency a fundoscopy is performed during routine follow-up is …	Multiple choice	Once a year; Once every two years; Once every three years; Not according to a standard schedule; Depending on patient's age; Other
**67**	Is the performance of an electroretinogram (ERG) part of routine follow-up?	Multiple choice	Yes; No, an ERG is only performed in case a patient develops signs of retinopathy; No, an ERG is never performed.
**68**	At what age does the performance of ERGs as part of routine follow-up start?	Open	
**69**	The frequency an ERG is performed during routine follow-up is …	Multiple choice	Once a year; Once every two years; Once every three years; Not according to a standard schedule; Depending on patient's age; Other
**70**	The frequency an ERG is performed in case a patient has developed signs of retinopathy, is …	Multiple choice	Once a year; Once every two years; Once every three years; Not according to a standard schedule; Depending on patient's age; Other
**71**	Are there any other tests you perform in the routine follow-up of retinopathy in patients with LCHAD or MTP deficiency? If yes, what other tests do you perform?	Open	
**72**	Are electrophysiological examinations (nerve conduction studies (ENG) and/or electromyography (EMG)) part of routine follow-up?	Multiple choice	Yes; No, electrophysiological examinations are only performed in case a patient develops signs or symptoms of neuropathy; No, electrophysiological examinations are never performed.
**73**	In case a patient develops signs of neuropathy, we perform...	Multiple choice	An electromyogram (EMG); Nerve conduction studies (ENG); Both an electromyogram and nerve conduction studies; Other
**74**	The frequency electrophysiogical examination is performed during follow-up of an established neuropathy is …	Multiple choice	Once a year; Once every two years; Once every three years; Not according to a standard schedule; Depending on patient's age; Other
**75**	As part of routine follow-up, we perform...	Multiple choice	An electromyogram (EMG); Nerve conduction studies (ENG); Both an electromyogram and nerve conduction studies; Other
**76**	At what age does electrophysiological examination start as part of routine follow-up?	Open	
**77**	The frequency electrophysiogical examination is performed during routine follow-up is...	Multiple choice	Once a year; Once every two years; Once every three years; Not according to a standard schedule; Depending on patient's age; Other
**78**	Are there any other tests you perform in the routine follow-up of neuropathy in patients with LCHAD or MTP deficiency? If yes, what other tests do you perform?	Open	

If ‘Other’ was chosen, respondents were able to fill in their answer in an open box
** Respondents only received relevant questions, for example: if a respondent answered ‘Yes’ to the question whether he or she performed an echocardiogram as part of routine follow-up, the question regarding frequency of follow-up was shown. If the respondent answered ‘No’, he or she skipped the question regarding frequency of follow-up.
