# Supplementary material for: Long-term monitoring of fatty acid oxidation defects: results from a MetabERN survey
Source: Orphanet J Rare Dis. 2024 Jan 20;19:21. doi: 10.1186/s13023-024-03024-0 (PMC10800038; doi:10.1186/s13023-024-03024-0)
Supplement: Supplementary file 2 — Additional file 2: An overview of the included countries and the number of included metabolic centres per country. [file 13023_2024_3024_MOESM2_ESM.pdf]

## Additional File 2: An overview of included countries and metabolic centres

| | Included countries | Number of respondents per country | Number of metabolic centres per country |
|---|---|---|---|
| 1. | France | 3 respondents | 3 different centres |
| 2. | Austria | 3 respondents | 3 different centres |
| 3. | Italy | 5 respondents | 5 different centres |
| 4. | Spain | 3 respondents | 3 different centres |
| 5. | Belgium | 2 respondents | At least 1 centre* |
| 6. | Germany | 7 respondents | 7 different centres |
| 7. | Turkey | 2 respondents | 2 different centres |
| 8. | Slovakia | 1 respondent | 1 centre |
| 9. | Latvia | 1 respondent | 1 centre |
| 10. | Norway | 1 respondent | 1 centre |
| 11. | Sweden | 2 respondents | 2 different centres |
| 12. | Netherlands | 2 respondents | 2 different centres |
| 13. | Denmark | 1 respondent | 1 centre |
| 14. | Slovenia | 1 respondent | 1 centre |
| 15. | United Kindom | 1 respondent | 1 centre |
| 16. | Ireland | 1 respondent | 1 centre |
| 17. | Unknown country | 1 respondent | 1 centre |

*Of 1 respondent, the metabolic centre was unknown
