# Supplementary material for: Long-term monitoring of fatty acid oxidation defects: results from a MetabERN survey
Source: Orphanet J Rare Dis. 2024 Jan 20;19:21. doi: 10.1186/s13023-024-03024-0 (PMC10800038; doi:10.1186/s13023-024-03024-0)
Supplement: Supplementary file 3 — Additional file 3: Overview of the reported follow-up frequencies in infants (A), toddlers (B), children (C), adolescents and adults (D). [file 13023_2024_3024_MOESM3_ESM.pdf]

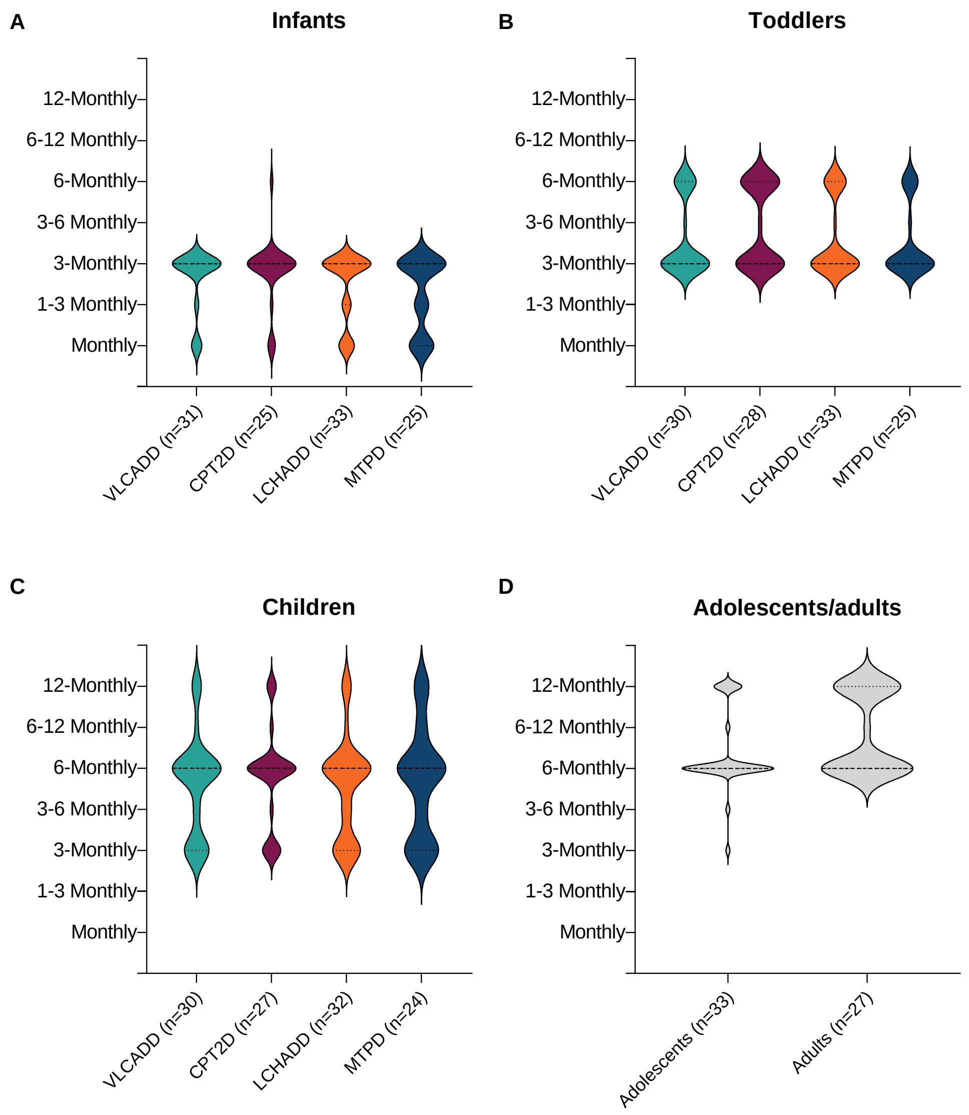

Additional File 3: Overview of the reported follow-up frequencies in infants (A), toddlers (B), children (C), adolescents and adults (D). For CPT2D infants, and all LCFAOD adolescents and adults, there was one respondent for whom follow-up frequency depended on disease severity (not shown in the graph).

Abbreviations: VLCADD: very long-chain acyl-CoA dehydrogenase deficiency, CPT2D: carnitine palmitoyltransferase 2 deficiency, LCHADD: long-chain 3-hydroxyacyl-CoA dehydrogenase deficiency, MTPD: mitochondrial trifunctional protein deficiency.
