# Supplementary material for: Long-term monitoring of fatty acid oxidation defects: results from a MetabERN survey
Source: Orphanet J Rare Dis. 2024 Jan 20;19:21. doi: 10.1186/s13023-024-03024-0 (PMC10800038; doi:10.1186/s13023-024-03024-0)
Supplement: Supplementary file 4 — Additional file 4: Reported muscle, cardiac, hepatic, retinopathy and neuropathy monitoring in the different centres. [file 13023_2024_3024_MOESM4_ESM.pdf]

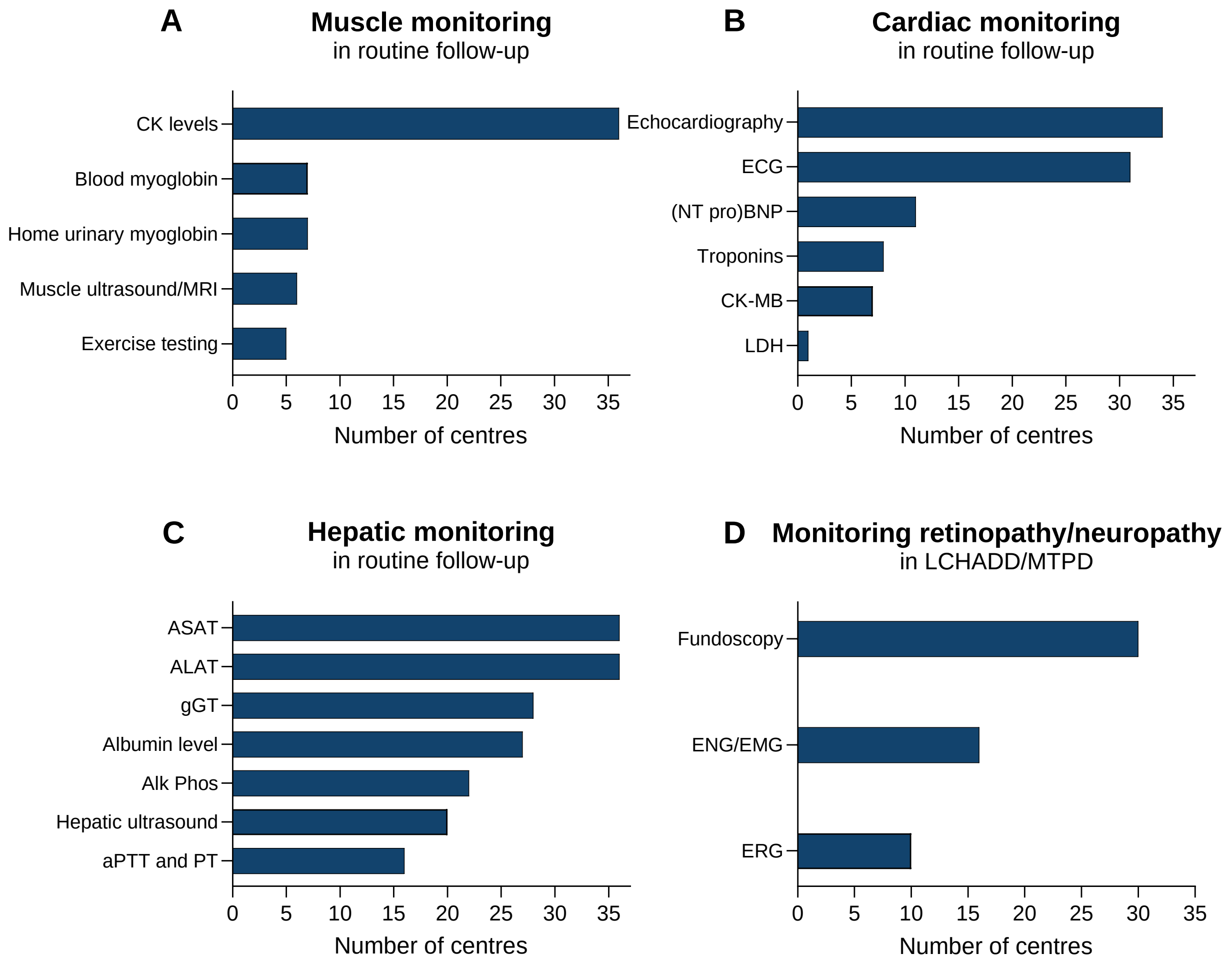

Additional File 4: Reported muscle, cardiac, hepatic, retinopathy and neuropathy monitoring in the different centres. A. Reported muscle monitoring in the different centres. B. Reported cardiac monitoring in the different centres. Of the 14 respondents measuring cardiac markers as part of the routine follow-up, 86% measured (NT pro)BNP, 57% troponins, 50% CK-MB, and 1% LDH. C. Reported hepatic monitoring in the different centres. Of the 36 respondents measuring hepatic markers as part of the routine follow-up, 100% measured ASAT and ALAT, 78% gGT, 75% albumin levels, 61% alkaline phosphatase, and 44% aPTT and PT. D. Reported long-term monitoring of retinopathy/neuropathy in the different centres. The graphs show the number of respondents performing the procedure.

Abbreviations: ECG: electrocardiogram, (NT pro)BNP: (N-terminal pro) B-type natriuretic peptide, CK(-MB): creatine kinase (–MB), LDH: lactate dehydrogenase, ASAT: aspartate aminotransaminase, ALAT: alanine aminotransferase, gGT: gamma-glutamyltransferase, Alk Phos: alkaline phosphatase, aPTT: activated partial thromboplastin time, PT: prothrombin time, MRI: magnetic resonance imaging, ENG: electroneurography, EMG: electromyography, ERG: electroretinography.
